# Supplementary material for: Decreased susceptibility of Plasmodium falciparum to both dihydroartemisinin and lumefantrine in northern Uganda
Source: Nat Commun. 2022 Oct 26;13:6353. doi: 10.1038/s41467-022-33873-x (PMC9605985; doi:10.1038/s41467-022-33873-x)
Supplement: Supplementary file 1 — Supplementary Tables [file 41467_2022_33873_MOESM1_ESM.pdf]

Supplemental Table 1. Genes targeted by the MIP panel.

Gene	Gene Name	Gene ID	RSA Subset[a]	Reference
amino acid transporter	AAT1	PF3D7_0629500	no	
amino acid transporter, putative	AAT2	PF3D7_1208400	no	
ABC transporter I family member 1, putative	ABCI3	PF3D7_0319700	no	
acetyl-CoA synthetase	ACS	PF3D7_0627800	no	
acyl-CoA synthetase	ACS10	PF3D7_0525100	yes	[1]
acyl-CoA synthetase	ACS11	PF3D7_1238800	no	
acetyl-CoA transporter, putative	ACT	PF3D7_1036800	no	
AP-2 complex subunit mu	AP2-MU	PF3D7_1218300	yes	[2]
AP-3 complex subunit delta, putative	AP3-D	PF3D7_0808100	no	
AP2 domain transcription factor, putative	ApiAP2	PF3D7_0613800	no	
ribosomal protein S10, apicoplast, putative	ARPS10	PF3D7_1460900	yes	[3, 4]
non-SERCA-type Ca2+ -transporting P-ATPase	ATP4	PF3D7_1211900	no	
calcium-transporting ATPase	ATP6	PF3D7_0106300	yes	[5, 6]
cyclic amine resistance locus protein	CARL	PF3D7_0321900	no	
coronin	Coronin	PF3D7_1251200	yes	[7, 8]
phenylalanine--tRNA ligase alpha subunit	cPheRS	PF3D7_0109800	no	
chloroquine resistance transporter	CRT	PF3D7_0709000	yes	[3]
cytochrome b	CYTB	PF3D7_MIT02300	no	
bifunctional dihydrofolate reductase-thymidylate synthase	DHFR-TS (DHFR)	PF3D7_0808100	no	
dihydroorotate dehydrogenase	DHODH	PF3D7_0603300	no	
dipeptidyl aminopeptidase 1	DPAP1	PF3D7_1116700	no	
elongation factor 2	eEF2	PF3D7_1451100	no	
DNA repair endonuclease XPF, putative	ERCC4	PF3D7_1368800	yes	[9]
3'-5' exonuclease, putative	EXO	PF3D7_1362500	no	
ferredoxin	FD	PF3D7_1318100	yes	[3, 10]
cysteine proteinase falcipain 1	FP1	PF3D7_1458000	yes	
cysteine proteinase falcipain 2a	FP2a	PF3D7_1115700	yes	[10, 11]
cysteine proteinase falcipain 2b	FP2b	PF3D7_1115300	yes	
cysteine proteinase falcipain 3	FP3	PF3D7_1115400	yes	
protein farnesyltransferase subunit beta	FTB	PF3D7_1147500	no	
GTP cyclohydrolase 1	GCH1	PF3D7_1224000	no	
GPI-anchored wall transfer protein 1, putative	GWT1	PF3D7_0615300	no	

Gene	Gene Name	Gene ID	RSA Subset[a]	Reference
histone deacetylase 1	HDAC1	PF3D7_0925700	no	
2-C-methyl-D-erythritol 4-phosphate cytidylyltransferase, putative	IspD	PF3D7_0106900	no	
kelch protein K10	Kelch10	PF3D7_1022600	yes	[12]
kelch protein K13	Kelch13 (K13)	PF3D7_1343700	yes	[11]
lysine--tRNA ligase	KRS1	PF3D7_1350100	no	
mitochondrial carrier protein, putative	MCP	PF3D7_1368700	no	
multidrug resistance protein 1	MDR1	PF3D7_0523000	no	
multidrug resistance protein 2	MDR2	PF3D7_1447900	yes	[3]
multidrug resistance-associated protein 1	MRP1	PF3D7_0112200	yes	[13]
multidrug resistance-associated protein 2	MRP2	PF3D7_1229100	no	
NLI interacting factor-like phosphatase, putative	NIF4 (pph)	PF3D7_1012700	yes	[3]
glycylpeptide N-tetradecanoyltransferase	NMT	PF3D7_1412800	no	
prodrug activation and resistance esterase	PARE	PF3D7_0709700	no	
phosphatidylinositol 4-kinase, putative	PI4K	PF3D7_0419900	yes	[12]
phosphatidylinositol 4-kinase beta	PI4KB	PF3D7_0509800	no	
cGMP-dependent protein kinase	PKG	PF3D7_1436600	no	
plasmepsin I	PM1	PF3D7_1407900	no	
plasmepsin II	PM2	PF3D7_1408000	no	
plasmepsin III	PM3	PF3D7_1408100	no	
plasmepsin IX	PMIX	PF3D7_1430200	no	
plasmepsin V	PMV	PF3D7_1323500	no	
plasmepsin X	PMX	PF3D7_0808200	no	
hydroxymethyldihydropterin pyrophosphokinase-dihydropteroate synthase	DHPS	PF3D7_0810800	no	
proline--tRNA ligase	PRS	PF3D7_1213800	no	
phosphoinositide-binding protein PX1	PX1 (PIB7)	PF3D7_0720700	yes	[3]
DNA repair protein RAD14, putative	RAD14	PF3D7_0710400	yes	[9]
26S proteasome regulatory subunit RPN10, putative	RPN10	PF3D7_0807800	no	
26S protease regulatory subunit 10B, putative	RPT4	PF3D7_1306400	no	
RuvB-like helicase 2	RUVB2	PF3D7_1106000	yes	[9]
CRAL/TRIO domain-containing protein, putative	Sec14	PF3D7_0626400	yes	[12]
transcriptional regulatory protein sir2a	Sir2A	PF3D7_1328800	no	
transcriptional regulatory protein sir2b	Sir2B	PF3D7_1451400	no	
subtilisin-like protease 1	Sub1	PF3D7_0507500	no	

Gene	Gene Name	Gene ID	RSA Subset[a]	Reference
ubiquitin carboxyl-terminal hydrolase 1, putative	UBP1	PF3D7_0104300	yes	[2, 12]
UDP-galactose transporter, putative	UGT	PF3D7_1113300	no	
V-type proton ATPase catalytic subunit A	VapA	PF3D7_1311900	no	
V-type proton ATPase subunit B	VapB	PF3D7_0406100	no	
V-type proton ATPase subunit D, putative	v-atpase	PF3D7_1341900	no	
patatin-like phospholipase, putative		PF3D7_0218600	no	
proteasome subunit alpha type 6, putative		PF3D7_0807500	no	
proteasome subunit beta type 1, putative		PF3D7_0518300	no	
proteasome subunit beta type 5		PF3D7_1011400	no	
proteasome subunit beta type 7, putative		PF3D7_1328100	no	
conserved Plasmodium protein, unknown function		PF3D7_1322700	yes	[3]
conserved Plasmodium protein, unknown function		PF3D7_1451200	yes	[3]
conserved Plasmodium protein, unknown function		PF3D7_1433800	yes	[7]
ubiquitin regulatory protein, putative		PF3D7_0808300	no	
V-type proton ATPase subunit a, putative		PF3D7_0806800	no	

[a]Proteins evaluated for associations with ex vivo RSA data.

Supplemental Table 2. Polymorphisms other than K13-propeller domain mutations that are associated with RSA survival. P-values were determined using two-sided Mann-Whitney Wilcoxon tests, without corrections for multiple comparisons.

Gene of interest	Locus	G'type	N	Median RSA Survival (%, (IQR))	p	K13/ G'type	N	Median RSA Survival (%, (IQR))	WT/ WT	WT/ Mut	469Y /WT	675V /WT	Loci used to make aggregate genotype variable
**PfCoronin PF3D7_1251200** coronin	aggregate of 8 loci	WT	38	2.3 (0.6-5.7)	ref	WT/WT	26	1.4 (0.5-5.2)	-	-	-	-	N507K, N553del, N553dup, D458N, E450K, P461L, P76S, V424I
		Mut	18	6.5 (2.0-11.4)	0.04	WT/Mut	9	2.6 (1.4-7.4)	0.31	-	-	-	
						469Y/WT	12	3.5 (2.2-5.5)	0.26	0.86	-	-	
						469Y/Mut	5	12.6 (7.6-14.3)	0.009	0.11	0.02	-	
						675V/WT	0	-	NA	NA	-	-	
						675V/Mut	4	5.3 (3.1-6.7)	0.50	0.94	-	NA	
**FP2A PF3D7_1115700** Cysteine Proteinase falcipain 2a	aggregate of 17 loci	WT	17	0.6 (0.0-3.2)	ref	WT/WT	11	0.0 (0.0-1.7)	-	-	-	-	D144N, Q15H, Q414E, G17E, K143E, K255G, S105N, S228T, S46L, S59F, T134K, Y113N, V51I, V51F, E248D, E249A, M245I
		Mut	47	3.2 (0.9-7.3)	0.02	WT/Mut	30	2.0 (0.8-7.1)	0.007	-	-	-	
						469Y/WT	3	8.4 (5.8-11.4)	0.02	0.16	-	-	
						469Y/Mut	15	3.9 (2.5-7.2)	0.004	0.27	0.41	-	
						675V/WT	3	0.0 (0.0-3.2)	1.00	0.18	-	-	
						675V/Mut	2	5.7 (4.9-6.5)	0.05	0.35	-	0.37	
**K13 PF3D7_1343700** kelch Protein K13	aggregate of 15 loci	WT	26	5.5 (2.3-7.3)	ref	WT/WT	13	5.1 (2.1-7.3)	-	-	-	-	R255K, N141_N142del, N141_N142dup, N142del, N142dup, N217H, Q271H, L258M, K189N, K189T, P96Q, S182T, T149S, T348I, E433D
		Mut	42	1.5 (0.4-3.4)	0.02	WT/Mut	31	1.0 (0.4-2.4)	0.02	-	-	-	
						469Y/WT	8	5.6 (3.4-9.5)	0.83	0.02	-	-	
						469Y/Mut	11	3.4 (2.0-7.2)	0.80	0.02	0.46	-	
						675V/WT	5	4.1 (0.0-6.5)	0.43	0.73	-	-	
						675V/Mut	0	-	NA	NA	-	NA	
**UBP1 PF3D7_0104300** ubiquitin carboxyl-terminal hydrolase 1, putative	N25_N26 del	Ref	16	1.3 (0.3-3.9)	ref	WT/WT	13	0.8 (0.0-2.2)	-	-	-	-	
		Mix	12	2.5 (1.1-5.0)	0.27	WT/Mut	10	2.4 (0.9-14.1)	0.05	-	-	-	
		Alt	8	10.5 (6.2-15.4)	0.003	469Y/WT	1	7.6 (7.6-7.6)	0.13	0.73	-	-	
		Mix/Alt	20	3.7 (1.6-13.2)	0.02	469Y/Mut	8	6.2 (3.2-13.2)	0.002	0.32	1.00	-	
						675V/WT	2	6.9 (6.7-7.1)	0.03	0.61	-	-	
						675V/Mut	1	0.0 (0.0-0.0)	0.31	0.18	-	0.67	
**PF3D7_1433800** conserved Protein	I1297V	Ref	27	5.8 (2.0-7.6)	ref	WT/WT	17	3.1 (0.8-7.4)	-	-	-	-	
		Mix	13	1.8 (0.4-3.4)	0.02	WT/Mut	13	1.7 (0.4-2.3)	0.07	-	-	-	
		Alt	11	2.5 (1.1-6.7)	0.33	469Y/WT	7	6.7 (4.8-10.5)	0.13	0.01	-	-	
		Mix/Alt	24	2.2 (0.4-5.4)	0.03	469Y/Mut	10	3.7 (2.5-7.0)	0.71	0.07	0.23	-	
						675V/WT	3	6.5 (5.3-6.9)	0.43	0.08	-	-	
						675V/Mut	1	0.0 (0.0-0.0)	0.11	0.26	-	0.50	

Abbreviations: G'type = genotype; ref = statistical reference; WT = major allele; Mut = minor allele; IQR = interquartile range

Supplemental Table 3. Polymorphisms associated with ex vivo chloroquine (CQ) sensitivities. P-values were determined using two-sided Mann-Whitney Wilcoxon tests, without corrections for multiple comparisons.

			Median CQ IC_{50} (nM, (N))				
Gene	Polymorphism		WT	Mix	Mutant	Mix/minor	p
CRT PF3D7_0709000	M74I	appears as haplotype	17.0 (614)	28.0 (42)	232.5 (26)	17.0 (68)	1.0×10^{-20}
	N75E						
	K76T						
	A220S		17.0 (250)	22.5 (18)	221.0 (11)	65.0 (29)	1.2×10^{-7}
	Q271E		17.0 (477)	25.0 (31)	232.5 (22)	130.0 (53)	6.8×10^{-17}
	R371I		17.0 (575)	22.0 (54)	232.5 (32)	51.5 (86)	4.4×10^{-18}
FP2a PF3D7_1115700	E115V		17.5 (770)	9.1 (3)	8.1 (1)	8.6 (4)	0.005
FP1 PF3D7_1458000	A42V		18.0 (703)	14.0 (28)	11.0 (7)	12.3 (35)	0.010
EXO PF3D7_1362500	N379_D382dup		19.0 (160)	4.7 (2)	8.1 (1)	5.7 (3)	0.007
MRP2 PF3D7_1229100	N1976S		18.0 (503)	6.3 (2)	7.9 (1)	7.9 (3)	0.008
	N1746del		18.8 (313)	17.0 (434)	6.1 (1)	17.0 (435)	0.005
UBP1 PF3D7_0104300	K1312_D1317dup		17.0 (634)	43.7 (2)	28.0 (3)	28.0 (5)	0.008
	K1314_D1317dup		17.4 (625)	12.7 (12)	8.5 (2)	12.7 (14)	0.014
ABCI3 PF3D7_0319700	K2673_D2676dup		17.0 (672)	40.0 (3)	99.0 (1)	69.5 (4)	0.011
PF3D7_0218600	R1446P		17.0 (584)	21.0 (25)	33.0 (6)	22.0 (31)	0.004
	I1859_N1861del		17.0 (711)	22.0 (14)	33.5 (2)	22.0 (16)	0.005
DPAP1 PF3D7_1116700	S292Y		17.0 (514)	20.0 (41)	26.0 (12)	21.0 (53)	0.004
PRS PF3D7_1213800	V186G		17.0 (776)	32.0 (7)	22.0 (1)	31.0 (8)	0.010
K13 PF3D7_1343700	C469Y		18.0 (745)	18.1 (14)	9.7 (10)	11.6 (24)	0.005
MDR1 PF3D7_0523000	D1246Y		17.0 (634)	20.0 (77)	21.0 (22)	20.0 (99)	0.010
ApiAP2 PF3D7_0613800	H887N		18.0 (565)	11.5 (8)	12.1 (4)	11.5 (12)	0.003
RAD14 PF3D7_0710400	T338K		18.0 (529)	17.0 (171)	13.9 (47)	16.0 (218)	0.009
FTB PF3D7_1147500	N302K		18.0 (737)	13.5 (16)	12.9 (2)	13.5 (18)	0.014

Supplemental Table 4. Polymorphisms associated with ex vivo monodesethylamodiaquine (MDAQ) sensitivities. P-values were determined using two-sided Mann-Whitney Wilcoxon tests, without corrections for multiple comparisons.

			Median MDAQ IC_{50} (nM, (N))				
Gene	Polymorphism		WT	Mix	Mutant	Mix/minor	p
CRT PF3D7_0709000	M74I	appears as haplotype	6.8 (463)	9 (13)	41.5 (4)	12 (17)	0.0003
	N75E						
	K76T						
	D24Y		7.1 (376)	6.4 (94)	4.4 (27)	6 (121)	0.011
	Q271E		6.9 (342)	8.1 (11)	67 (3)	10.5 (14)	0.01
Coronin PF3D7_1251200	E450K		6.9 (391)	12 (3)	202 (1)	18 (4)	0.003
PF3D7_0218600	N498_N506del		6.8 (216)	4.7 (10)	3.2 (8)	4.4 (18)	0.014
	N524_K525dup		7.2 (311)	6 (65)	5.3 (12)	6 (77)	0.007
ApiAP2 PF3D7_0613800	Q197E		5.8 (72)	7.1 (99)	7.3 (69)	7.2 (168)	0.011
	N2135T		6.8 (390)	8.2 (5)	12 (1)	9.3 (6)	0.01
PI4K PF3D7_0509800	G124D		8.5 (19)	3.9 (12)	6.2 (1)	3.9 (13)	0.015
UBP1 PF3D7_0104300	N25_N26del		6.5 (157)	6.9 (108)	8.2 (38)	7.3 (146)	0.007
	Y1307H		6.9 (475)	4.3 (9)	2.6 (1)	4 (10)	0.014
	N3094S		6.5 (262)	7.8 (37)	11.6 (3)	8.2 (40)	0.001
	H504Y		6.9 (505)	10 (7)	13.2 (2)	10 (9)	0.014
RAD14 PF3D7_0710400	I171M		7.4 (58)	7.3 (123)	6.7 (225)	6.7 (225)	0.012
FP2b PF3D7_1115300	H150N		6.7 (118)	7.3 (43)	8 (35)	7.6 (78)	0.009
	V157A		6.7 (129)	7.3 (40)	8.1 (27)	7.8 (67)	0.008
	T165M		6.7 (134)	7.4 (38)	8.1 (24)	7.8 (62)	0.003
ERCC4 PF3D7_1368800	K1149fs		6.8 (456)	8.2 (38)	7.9 (1)	8.1 (39)	0.01
AP2mu PF3D7_1218300	N233dup		6.7 (389)	7.5 (64)	7.7 (8)	7.5 (72)	0.002

Supplemental Table 5. Polymorphisms associated with ex vivo piperaquine (PQ) sensitivities. P-values were determined using two-sided Mann-Whitney Wilcoxon tests, without corrections for multiple comparisons.

		Median PQ IC_{50} (nM, (N))				
Gene	Polymorphism	WT	Mix	Mutant	Mix/minor	p
CRT PF3D7_0709000	A220S	5.1 (216)	3.5 (13)	2.3 (4)	2.7 (17)	0.012
ERCC4 PF3D7_1368800	L152I	4.9 (698)	10.5 (2)	13.9 (1)	11.0 (3)	0.013
PF3D7_1433800	E2377D	5.3 (22)	- (0)	2.0 (3)	2.0 (3)	0.014
	L2375V	5.3 (22)	- (0)	2.0 (3)	2.0 (3)	0.014
ABCI3 PF3D7_0319700	N1537K	4.9 (537)	10.3 (1)	12.5 (2)	12.0 (3)	0.012
DPAP1 PF3D7_1116700	L218V	4.7 (602)	7.0 (20)	11.0 (2)	7.6 (22)	0.007
MRP2 PF3D7_1229100	N935D	4.9 (537)	2.0 (2)	1.6 (1)	1.6 (3)	0.013
UBP1 PF3D7_0104300	F1739Y	4.9 (373)	1.8 (3)	1.6 (1)	1.7 (4)	0.003
	N3094S	4.3 (336)	5.4 (61)	7.3 (3)	5.5 (64)	0.008
Sir2B PF3D7_1451400	N488_Y492del	4.8 (663)	8.6 (14)	6.2 (1)	8.2 (15)	0.002
PF3D7_0218600	N501_N506del	5.0 (273)	3.6 (41)	2.4 (4)	3.4 (45)	0.007
	M632I	5.0 (625)	4.1 (49)	2.4 (2)	4.0 (51)	0.01
PF3D7_0808100	E1534K	4.9 (625)	2.3 (6)	2.7 (2)	2.3 (8)	0.005
PF3D7_0806800	N775_N778del	4.8 (625)	6.6 (23)	6.1 (1)	6.5 (24)	0.007

Supplemental Table 6. Polymorphisms associated with ex vivo mefloquine (MQ) sensitivities. P-values were determined using two-sided Mann-Whitney Wilcoxon tests, without corrections for multiple comparisons.

		Median MQ IC_{50} (nM, (N))				
Gene	Polymorphism	WT	Mix	Mutant	Mix/minor	p
MDR1 PF3D7_0523000	Phe938Tyr	10 (604)	6.9 (24)	7.1 (5)	7.1 (29)	0.001
CRT PF3D7_0709000	Gln271Glu	10.1 (415)	7.9 (24)	6.7 (9)	7.2 (33)	0.001
	Arg371Ile	10.4 (508)	8.6 (38)	8 (16)	8.4 (54)	0.013
PF3D7_0806800	Gly918_Gly919dup	10 (621)	13 (9)	20.5 (2)	14 (11)	0.006
PR5 PF3D7_1213800	Asn223_Asn224dup	11 (484)	8.5 (140)	4.5 (1)	8.5 (141)	0.001
ERCC4 PF3D7_1368800	Gln1569Glu	11 (276)	7.4 (60)	9.5 (49)	8.4 (109)	0.012
Sir2b PF3D7_1451400	Asn462dup	11 (570)	8.4 (63)	7.8 (13)	8.1 (76)	0.004
DPAP1 PF3D7_1116700	Lys216Thr	10 (592)	8.4 (7)	3.1 (3)	5.3 (10)	0.013
UBP1 PF3D7_0104300	Asn452fs	9.8 (438)	12 (62)	10.5 (10)	11 (72)	0.010

Supplemental Table 7. Polymorphisms associated with ex vivo pyronaridine (PND) sensitivities. P-values were determined using two-sided Mann-Whitney Wilcoxon tests, without corrections for multiple comparisons.

		Median PND IC_{50} (nM, (N))				
Gene	Polymorphism	WT	Mix	Mutant	Mix/minor	p
EXO PF3D7_1362500	N379_D382dup	1.3 (113)	0.2 (2)	0.6 (1)	0.3 (3)	0.012
K13 PF3D7_1343700	A675V	1.0 (661)	1.9 (9)	1.8 (3)	1.9 (12)	0.008
ApiAP2 PF3D7_0613800	R1034C	0.9 (99)	1.1 (280)	1.0 (291)	1.0 (291)	0.014
	N1722dup	1.0 (515)	1.5 (99)	1.0 (16)	1.4 (115)	0.001
	N2135T	1.0 (376)	2.7 (5)	1.1 (1)	2.4 (6)	0.011
ACS10 PF3D7_0525100	A637T	1.0 (515)	1.1 (114)	1.4 (27)	1.1 (141)	0.012
ACS PF3D7_0627800	S743A	1.0 (632)	0.5 (8)	0.4 (2)	0.4 (10)	0.010
PF3D7_0806800	D873_D874del	0.9 (190)	1.1 (338)	1.0 (123)	1.1 (461)	0.006
FP2a PF3D7_1115700	Q15His	1.0 (466)	1.2 (78)	1.8 (18)	1.4 (96)	0.002
FP1 PF3D7_1458000	E162D	1.0 (625)	0.2 (4)	0.1 (1)	0.2 (5)	0.014
AP2mu PF3D7_1218300	N233dup	1.0 (487)	1.4 (85)	1.0 (10)	1.3 (95)	0.003
ATP4 PF3D7_1211900	G223S	1.0 (530)	1.3 (121)	1.1 (19)	1.2 (140)	0.010
MRP2 PF3D7_1229100	N646D	1.3 (41)	1.2 (151)	0.9 (197)	0.9 (197)	0.007
cPheRS PF3D7_0109800	G41_D43dup	1.0 (610)	1.4 (27)	2.7 (6)	1.7 (33)	0.007
UBP1 PF3D7_0104300	N233_Tyr234insNSINNSIN	0.9 (196)	1.2 (217)	1.0 (124)	1.1 (341)	0.002
	N452fs	1.0 (452)	1.6 (65)	1.1 (11)	1.3 (76)	0.001
	K1063_N1064del	1.0 (570)	1.4 (52)	1.0 (12)	1.3 (64)	0.005
	E1929K	1.0 (469)	1.2 (64)	1.0 (20)	1.2 (84)	0.014

Supplemental Table 8. Polymorphisms associated with ex vivo dihydroartemisinin (DHA) sensitivities.
P-values were determined using two-sided Mann-Whitney Wilcoxon tests, without corrections for multiple comparisons.

		Median DHA IC_{50} (nM, (N))				
Gene	Polymorphism	WT	Mix	Mutant	Mix/minor	p
K13 PF3D7_1343700	C469Y	1.5 (664)	2.3 (14)	2.4 (10)	10 (2.4)	0.002
	T348I	1.5 (519)	1.2 (7)	1 (5)	5 (1)	0.013
MDR1 PF3D7_0523000	D1246Y	1.5 (563)	1.2 (65)	1.3 (20)	20 (1.2)	0.003
	Y500N	1.5 (513)	2.6 (5)	4.7 (1)	1 (3.7)	0.001
MDR2 PF3D7_1447900	I492V	1.6 (280)	1.4 (229)	1.5 (123)	123 (1.5)	0.002
PM1 PF3D7_1407900	V20L	1.5 (480)	3.6 (2)	3.5 (1)	1 (3.5)	0.011
CRT PF3D7_0709000	A220S	1.5 (213)	1.1 (12)	1 (5)	5 (1)	0.013
	R371I	1.5 (521)	1.2 (41)	1.2 (20)	20 (1.2)	0.011
	Q271E	1.6 (429)	1.1 (25)	1 (12)	12 (1.1)	0.0003
CYTB PF3D7_MIT02300	F37C	1.5 (501)	4.1 (2)	4.7 (1)	1 (4.7)	0.013
DHODH	L28F	1.5 (559)	2.6 (5)	3.7 (1)	1 (2.6)	0.013
PF3D7_0218600	N1323_N1325dup	1.6 (308)	1.5 (280)	0.8 (4)	4 (1.4)	0.001
	N1324_N1325dup	1.6 (270)	1.5 (318)	0.7 (4)	4 (1.5)	0.004
	D945_D950dup	1.6 (335)	1.4 (194)	1.5 (69)	69 (1.4)	0.014
ApiAP2 PF3D7_0613800	V175A	1.5 (584)	1.2 (19)	1.1 (4)	4 (1.2)	0.007
	N1258_N1277del	1.5 (346)	0.9 (9)	1.5 (4)	4 (1)	0.015
	N2381_N2382del	1.6 (430)	1.5 (229)	1.1 (4)	4 (1.5)	0.012
	D1144N	1.5 (635)	1 (20)	1 (1)	1 (1)	0.014
RAD14 PF3D7_0710400	N125del	1.6 (382)	1.3 (95)	1.5 (34)	34 (1.3)	0.002
FP2a PF3D7_1115700	K255R	1.4 (21)	1.4 (166)	1.6 (399)	399 (1.6)	0.013
ABCI3 PF3D7_0319700	A1718V	1.6 (493)	0.3 (2)	0.7 (1)	1 (0.4)	0.006
FTB PF3D7_1147500	H360L	1.5 (642)	0.7 (3)	0.9 (3)	3 (0.9)	0.006
UBP1 PF3D7_0104300	R1133S	1.4 (92)	1.5 (211)	1.7 (244)	244 (1.7)	0.009

Supplemental Table 9. Design of drug resistance MIP panel. The design of each MIP used in the panel is provided in a .xlsx file at https://github.com/PJRosenthalLab/2022_Tumwebaze_NatCom/blob/main/Supplemental_Table_9_Design%20of%20drug%20resistance%20MIP%20panel.xlsx

Works Cited

1. Siddiqui, F.A., et al. Role of *Plasmodium falciparum* kelch 13 protein mutations in *P. falciparum* populations from northeastern Myanmar in mediating artemisinin resistance. *mBio* **11**, e01134-19 (2020).
2. Henriques, G., et al. Directional selection at the *pfmdr1*, *pfcrt*, *pfubp1*, and *pfap2mu* loci of *Plasmodium falciparum* in Kenyan children treated with ACT. *J Infect Dis* **210**, 2001-2008 (2014).
3. Miotto, O., et al. Genetic architecture of artemisinin-resistant *Plasmodium falciparum*. *Nat Genet* **47**, 226-234 (2015).
4. Button-Simons, K.A., et al. The power and promise of genetic mapping from *Plasmodium falciparum* crosses utilizing human liver-chimeric mice. *Commun Biol* **4**, 734 (2021).
5. Uhlemann, A.C., et al. A single amino acid residue can determine the sensitivity of SERCAs to artemisinins. *Nat Struct Mol Biol* **12**, 628-629 (2005).
6. Jambou, R., et al. Resistance of *Plasmodium falciparum* field isolates to in-vitro artemether and point mutations of the SERCA-type PfATPase6. *Lancet* **366**, 1960-1963 (2005).
7. Demas, A.R., et al. Mutations in *Plasmodium falciparum* actin-binding protein coronin confer reduced artemisinin susceptibility. *Proc Natl Acad Sci U S A* **115**, 12799-12804 (2018).
8. Sharma, A.I., et al. Genetic background and PfKelch13 affect artemisinin susceptibility of PfCoronin mutants in *Plasmodium falciparum*. *PLoS Genet* **16**, e1009266 (2020).
9. Xiong, A., et al. K13-mediated reduced susceptibility to artemisinin in *Plasmodium falciparum* is overlaid on a trait of enhanced DNA damage repair. *Cell Rep* **32**, 107996 (2020).
10. Takala-Harrison, S., et al. Independent emergence of artemisinin resistance mutations among *Plasmodium falciparum* in Southeast Asia. *J Infect Dis* **211**, 670-679 (2015).
11. Ariey, F., et al. A molecular marker of artemisinin-resistant *Plasmodium falciparum* malaria. *Nature*,. **505**, 50-55 (2014).
12. Cerqueira, G.C., et al. Longitudinal genomic surveillance of *Plasmodium falciparum* malaria parasites reveals complex genomic architecture of emerging artemisinin resistance. *Genome Biol* **18**, 78 (2017).
13. Dahlstrom, S., et al. *Plasmodium falciparum* multidrug resistance protein 1 and artemisinin-based combination therapy in Africa. *J Infect Dis* **200**, 1456-1464 (2009).
