# Supplementary material for: Decreased susceptibility of Plasmodium falciparum to both dihydroartemisinin and lumefantrine in northern Uganda
Source: Nat Commun. 2022 Oct 26;13:6353. doi: 10.1038/s41467-022-33873-x (PMC9605985; doi:10.1038/s41467-022-33873-x)
Supplement: Supplementary file 3 — Reporting Summary [file 41467_2022_33873_MOESM3_ESM.pdf]

nature portfolio

Corresponding author(s): Rosenthal, Philip J.

Last updated by author(s): Sep 28, 2022

Reporting Summary

Nature Portfolio wishes to improve the reproducibility of the work that we publish. This form provides structure for consistency and transparency in reporting. For further information on Nature Portfolio policies, see our Editorial Policies and the Editorial Policy Checklist.

Statistics

For all statistical analyses, confirm that the following items are present in the figure legend, table legend, main text, or Methods section.

n/a	Confirmed	
☐	☒	The exact sample size (*n*) for each experimental group/condition, given as a discrete number and unit of measurement
☐	☒	A statement on whether measurements were taken from distinct samples or whether the same sample was measured repeatedly
☐	☒	The statistical test(s) used AND whether they are one- or two-sided<br>*Only common tests should be described solely by name; describe more complex techniques in the Methods section.*
☒	☐	A description of all covariates tested
☐	☒	A description of any assumptions or corrections, such as tests of normality and adjustment for multiple comparisons
☐	☒	A full description of the statistical parameters including central tendency (e.g. means) or other basic estimates (e.g. regression coefficient) AND variation (e.g. standard deviation) or associated estimates of uncertainty (e.g. confidence intervals)
☐	☒	For null hypothesis testing, the test statistic (e.g. *F*, *t*, *r*) with confidence intervals, effect sizes, degrees of freedom and *P* value noted<br>*Give P values as exact values whenever suitable.*
☒	☐	For Bayesian analysis, information on the choice of priors and Markov chain Monte Carlo settings
☒	☐	For hierarchical and complex designs, identification of the appropriate level for tests and full reporting of outcomes
☒	☐	Estimates of effect sizes (e.g. Cohen's *d*, Pearson's *r*), indicating how they were calculated

Our web collection on statistics for biologists contains articles on many of the points above.

Software and code

Policy information about availability of computer code

Data collection	Data was collected using case record forms and then entered into Excel software (version 2202). Molecular inversion probes were designed using MIPTools software (version 0.19.12.13). IC50 values were generated using Prism (version 9.0). All additional analyses were done in R (version 3.4.4). No code was utilized for data collection.
Data analysis	All analyses were performed in R, as described in Methods.

For manuscripts utilizing custom algorithms or software that are central to the research but not yet described in published literature, software must be made available to editors and reviewers. We strongly encourage code deposition in a community repository (e.g. GitHub). See the Nature Portfolio guidelines for submitting code & software for further information.

Data

Policy information about availability of data

All manuscripts must include a data availability statement. This statement should provide the following information, where applicable:

- Accession codes, unique identifiers, or web links for publicly available datasets
- A description of any restrictions on data availability
- For clinical datasets or third party data, please ensure that the statement adheres to our policy

Data availability. All relevant data are available from the corresponding author upon request. Sequence data are available in the National Center for Biotechnology Information (NCBI) Sequence Read Archive (accession number PRJNA850445).

March 2021

Human research participants

Policy information about studies involving human research participants and Sex and Gender in Research.

Reporting on sex and gender	We recruited subjects with malaria without consideration of gender. Gender composition is provided in Table 1.
Population characteristics	This information is provided in Table 1.
Recruitment	Patients were recruited by identifying positive blood films among samples tested in study clinics. Isolates were collected from consenting patients aged 6 months or older presenting at the sites with clinical symptoms of malaria, a positive Giemsa-stained blood film for P. falciparum, and no signs of severe disease. Written informed consent was obtained from all participants. We do not anticipate that there was any self-selection bias or other biases, as there was no benefit to subjects and minimal inconvenience to subjects based on participation.
Ethics oversight	The studies were approved by the Makerere University Research and Ethics Committee, the Uganda National Council for Science and Technology, and the University of California, San Francisco Committee on Human Research.

Note that full information on the approval of the study protocol must also be provided in the manuscript.

Field-specific reporting

Please select the one below that is the best fit for your research. If you are not sure, read the appropriate sections before making your selection.

☒ Life sciences ☐ Behavioural & social sciences ☐ Ecological, evolutionary & environmental sciences

For a reference copy of the document with all sections, see nature.com/documents/nr-reporting-summary-flat.pdf

Life sciences study design

All studies must disclose on these points even when the disclosure is negative.

Sample size	Sample size, necessarily limited by cost and logistical constraints, was determined by available samples for comparison of isolates from eastern and northern Uganda in 2021 and considerations of all isolates with ex vivo characterization from 2015-21.
Data exclusions	Patients reporting use of antimalarial treatment within the previous 30 days or with evidence of an infection with other Plasmodium species were excluded.
Replication	Replicates were not possible for ex vivo studies and were not practical for genomic studies due to time and cost constraints. Controls were included to assure data quality. Ex vivo assays, which made up a good part of the data, necessarily cannot be replicated (they are a one-time assessment of drug susceptibility performed soon after sample collection). For genomic data, reads were reported to reach adequate data quality, as described in Methods (Individual genotypes were assigned for polymorphic sites that were covered by a minimum of 10 unique molecular identifiers (UMIs) and variants were required to have a within sample allele count ≥3 UMIs for alternate alleles and ≥2 UMIs for reference alleles). For samples in which sequencing was poor quality or unsuccessful, sequencing runs were repeated. Control DNA was included in all sequencing runs to assure data quality.
Randomization	Randomization is not relevant for this study, which offers descriptive data concerning P. falciparum isolates from patients presenting with malaria in Uganda. All samples were handled in the same manner. It is not appropriate to randomize subjects for a non-interventional study in which the goal is to manage each subject and each sample in the same manner.
Blinding	This was not a blinded study. It was a descriptive study concerning P. falciparum isolates from patients presenting with malaria in Uganda. All samples were handled in the same manner. It is not appropriate to incorporate blinding into a study in which the goal is to manage each subject and each sample in the same manner.

Reporting for specific materials, systems and methods

We require information from authors about some types of materials, experimental systems and methods used in many studies. Here, indicate whether each material, system or method listed is relevant to your study. If you are not sure if a list item applies to your research, read the appropriate section before selecting a response.

Materials & experimental systems

n/a	Involved in the study
☒	☐ Antibodies
☐	☒ Eukaryotic cell lines
☒	☐ Palaeontology and archaeology
☒	☐ Animals and other organisms
☒	☐ Clinical data
☒	☐ Dual use research of concern

Methods

n/a	Involved in the study
☒	☐ ChIP-seq
☒	☐ Flow cytometry
☒	☐ MRI-based neuroimaging

Eukaryotic cell lines

Policy information about cell lines and Sex and Gender in Research

Cell line source(s)	The P. falciparum laboratory strains 3D7 (MRA-102) and Dd2 (MRA-156) were obtained from BEI Resources, Manassas, VA, USA.
Authentication	Cell lines were authenticated by BEI resources. In addition, we authenticated the assays with each drug susceptibility assay, assuring that results for multiple drugs were similar to those published for the reference strains.
Mycoplasma contamination	Cell lines were not tested for mycoplasma contamination.
Commonly misidentified lines (See ICLAC register)	No commonly misidentified lines were used.
